# Supplementary material for: AlphaFold-guided phylogenetic analyses suggest surprising heterogeneity in metazoan replication origin licensing mechanisms
Source: EMBO J. 2025 Nov 27;45(1):310–33. doi: 10.1038/s44318-025-00628-5 (PMC12759066; doi:10.1038/s44318-025-00628-5)
Supplement: Supplementary file 1 — Appendix [file 44318_2025_628_MOESM1_ESM.pdf]

Appendix

AlphaFold-guided structural phylogenetic analysis suggests surprising heterogeneity in metazoan origin licensing mechanisms

Olivia Hunker and Franziska Bleichert

Department of Molecular Biophysics and Biochemistry, Yale University, New Haven, CT, USA

Correspondence: Franziska Bleichert
franziska.bleichert@yale.edu

Table of Contents:

Appendix Figure S1 .... Page 2
Appendix Figure S2 .... Page 3

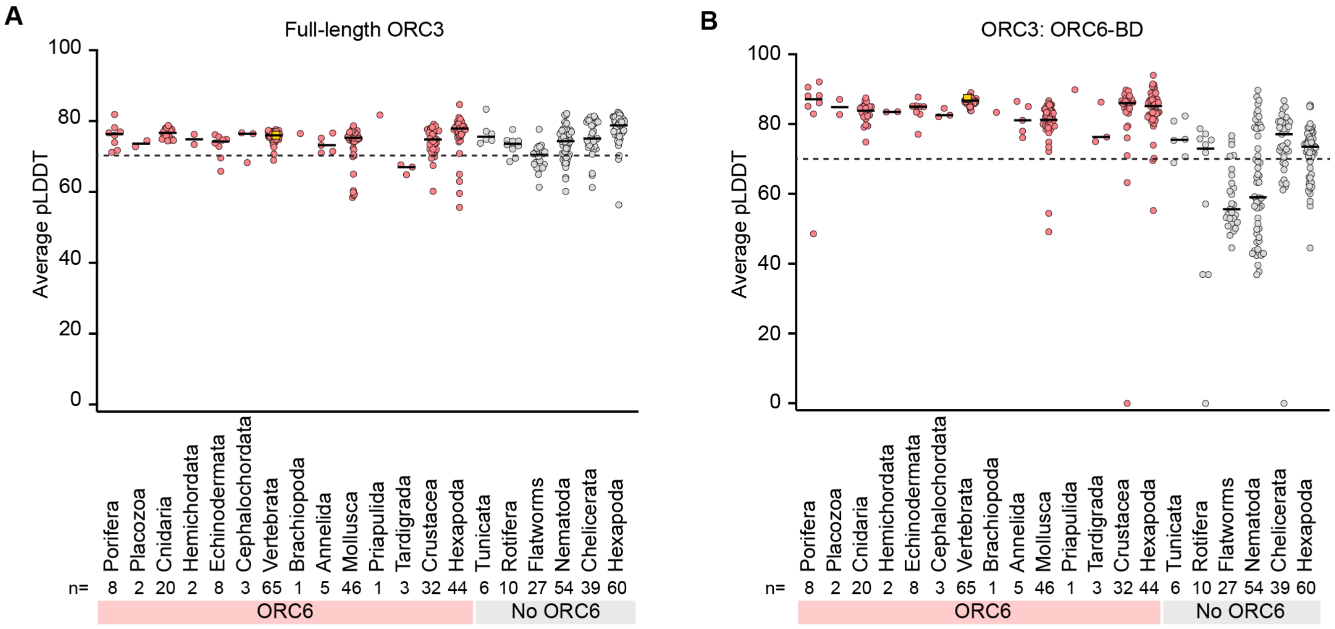

Appendix Figure S1. AlphaFold2 predictions of ORC3 orthologs are confident across most phyla. Average pLDDT scores from ORC3 ortholog AlphaFold2 predictions by individual phyla or subphyla for **A)** the entire ORC3 protein and **B)** the ORC6-BD region. Solid black lines represent medians. Dotted lines mark pLDDT = 70.

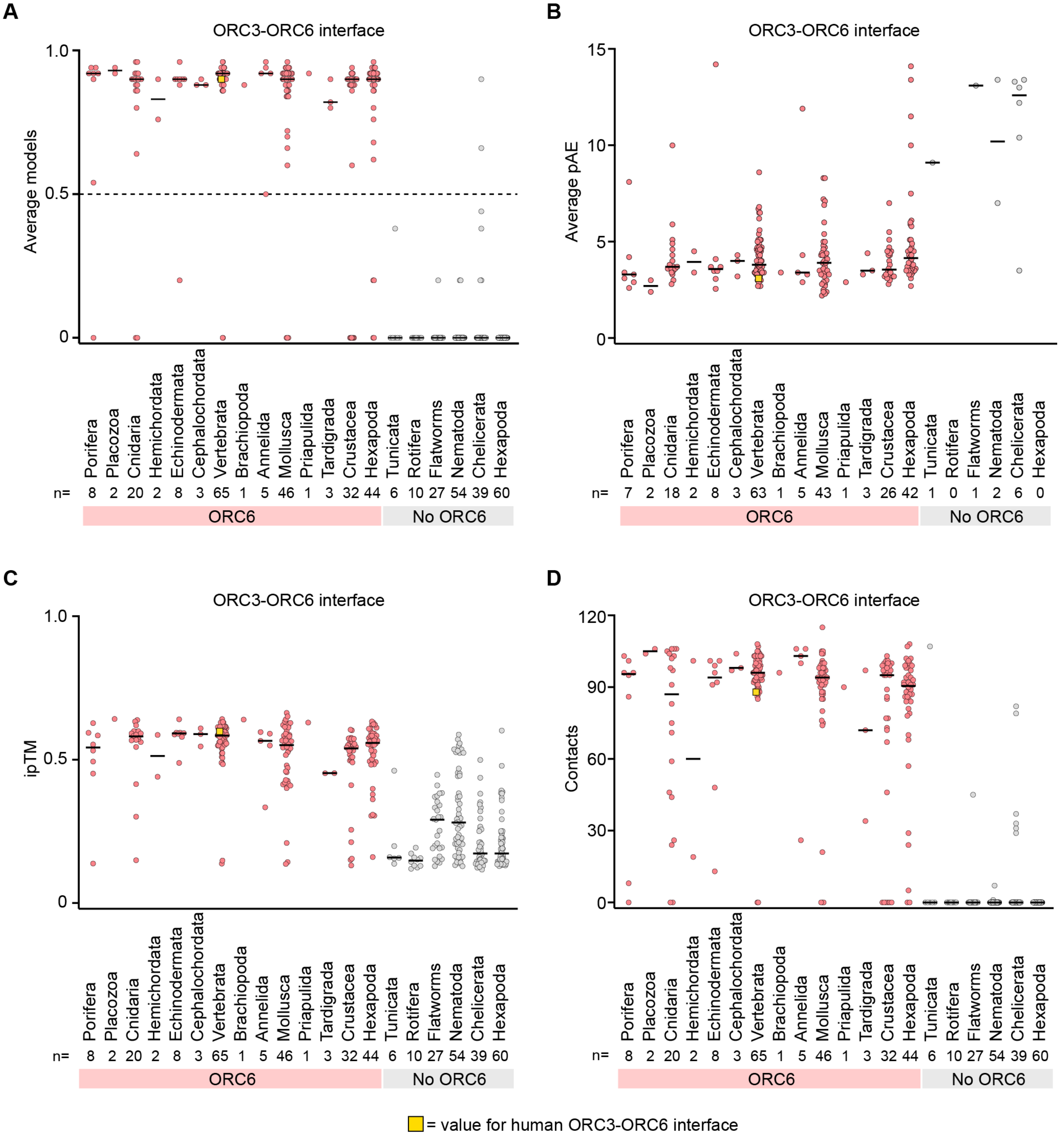

Appendix Figure S2. Confidence statistics for AlphaFold2 Multimer predictions of human ORC6 and metazoan ORC3 interactions by taxonomic group. A) *Average models* scores for canonical ORC3-ORC6 interfaces in AlphaFold2 predictions for each phylum/ subphylum. Dotted black line at *average models* = 0.5 marks the confidence cut-off. **B)** Average interface pAE scores of the canonical ORC3-ORC6 interface for each phylum/subphylum. If no interface was formed, pAE could not be calculated and no data is shown in these instances. **C)** ipTM scores for ORC3-ORC6 AlphaFold2 Multimer predictions for each phylum/subphylum. **D)** Number of contacts in the canonical ORC3-ORC6 interface in AlphaFold2 Multimer predictions

for each phylum/subphylum. Solid black lines in **A**-**D** represent medians. Values of 0 in **A** and **D** indicate that no canonical ORC3-ORC6 interface was predicted.
