# Supplementary material for: AlphaFold-guided phylogenetic analyses suggest surprising heterogeneity in metazoan replication origin licensing mechanisms
Source: EMBO J. 2025 Nov 27;45(1):310–33. doi: 10.1038/s44318-025-00628-5 (PMC12759066; doi:10.1038/s44318-025-00628-5)
Supplement: Supplementary file 2 — Table EV1 [file 44318_2025_628_MOESM2_ESM.docx]

**Table EV1. ORC1-6 orthologs found for species included in the ORC3 AlphaFold dataset.**

| **Phylum/Subphylum** | **ORC1** | **ORC2** | **ORC3** | **ORC4** | **ORC5** | **ORC6** | **Total species** |
| --- | --- | --- | --- | --- | --- | --- | --- |
| Porifera | 8 | 8 | 8 | 8 | 7 | 6 | 8 |
| Placozoa | 2 | 2 | 2 | 2 | 2 | 2 | 2 |
| Ctenophora | 1 | 1 | 0 | 1 | 1 | 1 | 1 |
| Cnidaria | 20 | 20 | 20 | 20 | 20 | 15 | 20 |
| Hemichordata | 2 | 2 | 2 | 2 | 2 | 2 | 2 |
| Echinodermata | 8 | 8 | 8 | 8 | 8 | 7 | 8 |
| Cephalochordata | 3 | 3 | 3 | 3 | 3 | 3 | 3 |
| Tunicata | 6 | 6 | 6 | 5 | 6 | 0 | 6 |
| Vertebrata | 64 | 64 | 65 | 65 | 65 | 53 | 65 |
| Rotifera | 9 | 10 | 10 | 10 | 8 | 0 | 10 |
| Platyhelminthes | 27 | 27 | 27 | 27 | 27 | 0 | 27 |
| Brachiopoda | 1 | 1 | 1 | 1 | 1 | 1 | 1 |
| Annelida | 5 | 5 | 5 | 5 | 5 | 4 | 5 |
| Mollusca | 46 | 46 | 46 | 46 | 46 | 35 | 46 |
| Priapulida | 1 | 1 | 1 | 1 | 1 | 1 | 1 |
| Nematoda | 54 | 54 | 54 | 46 | 54 | 0 | 54 |
| Tardigrada | 3 | 3 | 3 | 3 | 3 | 3 | 3 |
| Crustacea | 29 | 32 | 32 | 32 | 32 | 24 | 32 |
| Chelicerata | 39 | 39 | 39 | 39 | 39 | 0 | 39 |
| Hexapoda | 104 | 104 | 104 | 100 | 92 | 36 | 104 |
