# Supplementary material for: AlphaFold-guided phylogenetic analyses suggest surprising heterogeneity in metazoan replication origin licensing mechanisms
Source: EMBO J. 2025 Nov 27;45(1):310–33. doi: 10.1038/s44318-025-00628-5 (PMC12759066; doi:10.1038/s44318-025-00628-5)
Supplement: Supplementary file 10 — Expanded View Figures [file 44318_2025_628_MOESM10_ESM.pdf]

Expanded View Figures

Figure EV1. ORC6 conservation is variable across Metazoa and within the *Hexapoda* subphylum.

(**A**) Overview of proposed human origin licensing pathways. (**B**) Comparison of human ORC3 (PDB 7JPO (Jaremko et al, 2020)) and *C. elegans* ORC3 (AlphaFold database, accession number Q95Y69), demonstrating loss of the entire ORC6-BD region in *C. elegans* ORC3. (**C**, **D**) Percent sequence identity of metazoan ORC6-BDs as compared to the human ORC6-BD. Species without an ORC6 ortholog have greater sequence-level variation from the human ORC6-BD than species with an ORC6 ortholog. A comparison of percent identities for species with and without an ORC6 ortholog (in **C**) and for each taxonomic group surveyed (in **D**) is shown. Solid black lines indicate median values. (**E**) Multiple ORC3 protein sequence alignment shows variable conservation of the ORC6-BD in several hexapod orders. (**F**) Phylogenetic tree summarizing the presence or absence of ORC6 orthologs across hexapoda. All groups have ORC1-5 orthologs. Tree construction is based on established phylogeny (Misof et al, 2014) and distances depicted are arbitrary.

© The Author(s)

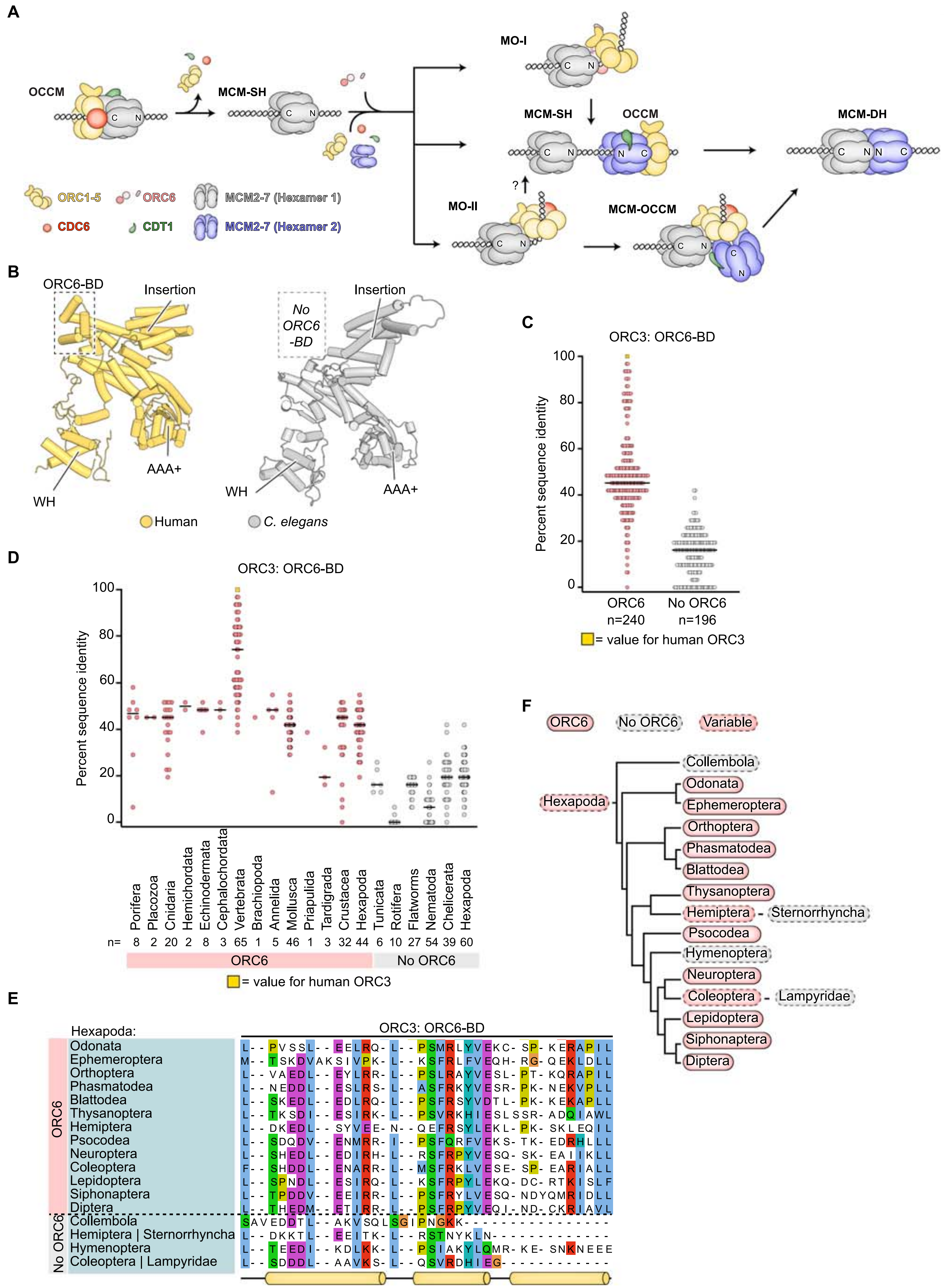
A
OCCM
MCM-SH
MO-I
MCM-SH
OCCM
MCM-DH
MO-II
MCM-OCCM
?
ORC1-5
ORC6
MCM2-7 (Hexamer 1)
CDC6
CDT1
MCM2-7 (Hexamer 2)
B
ORC6-BD
Insertion
No ORC6 -BD
Insertion
WH
AAA+
WH
AAA+
Human
C. elegans
C
ORC3: ORC6-BD
Percent sequence identity
ORC6 n=240
No ORC6 n=196
= value for human ORC3
D
ORC3: ORC6-BD
Percent sequence identity
Porifera
Placozoa
Cnidaria
Hemichordata
Echinodermata
Cephalochordata
Vertebrata
Brachiopoda
Annelida
Mollusca
Priapulida
Tardigrada
Crustacea
Hexapoda
Tunicata
Rotifera
Flatworms
Nematoda
Chelicerata
Hexapoda
n= 8 2 20 2 8 3 65 1 5 46 1 3 32 44 6 10 27 54 39 60
ORC6
No ORC6
= value for human ORC3
E
Hexapoda:
ORC3: ORC6-BD
ORC6
Odonata
Ephemeroptera
Orthoptera
Phasmatodea
Blattodea
Thysanoptera
Hemiptera
Psocodea
Neuroptera
Coleoptera
Lepidoptera
Siphonaptera
Diptera
No ORC6
Collembola
Hemiptera | Sternorrhyncha
Hymenoptera
Coleoptera | Lampyridae
F
ORC6
No ORC6
Variable
Hexapoda
Collembola
Odonata
Ephemeroptera
Orthoptera
Phasmatodea
Blattodea
Thysanoptera
Hemiptera
Sternorrhyncha
Psocodea
Hymenoptera
Neuroptera
Coleoptera
Lampyridae
Lepidoptera
Siphonaptera
Diptera

© The Author(s)

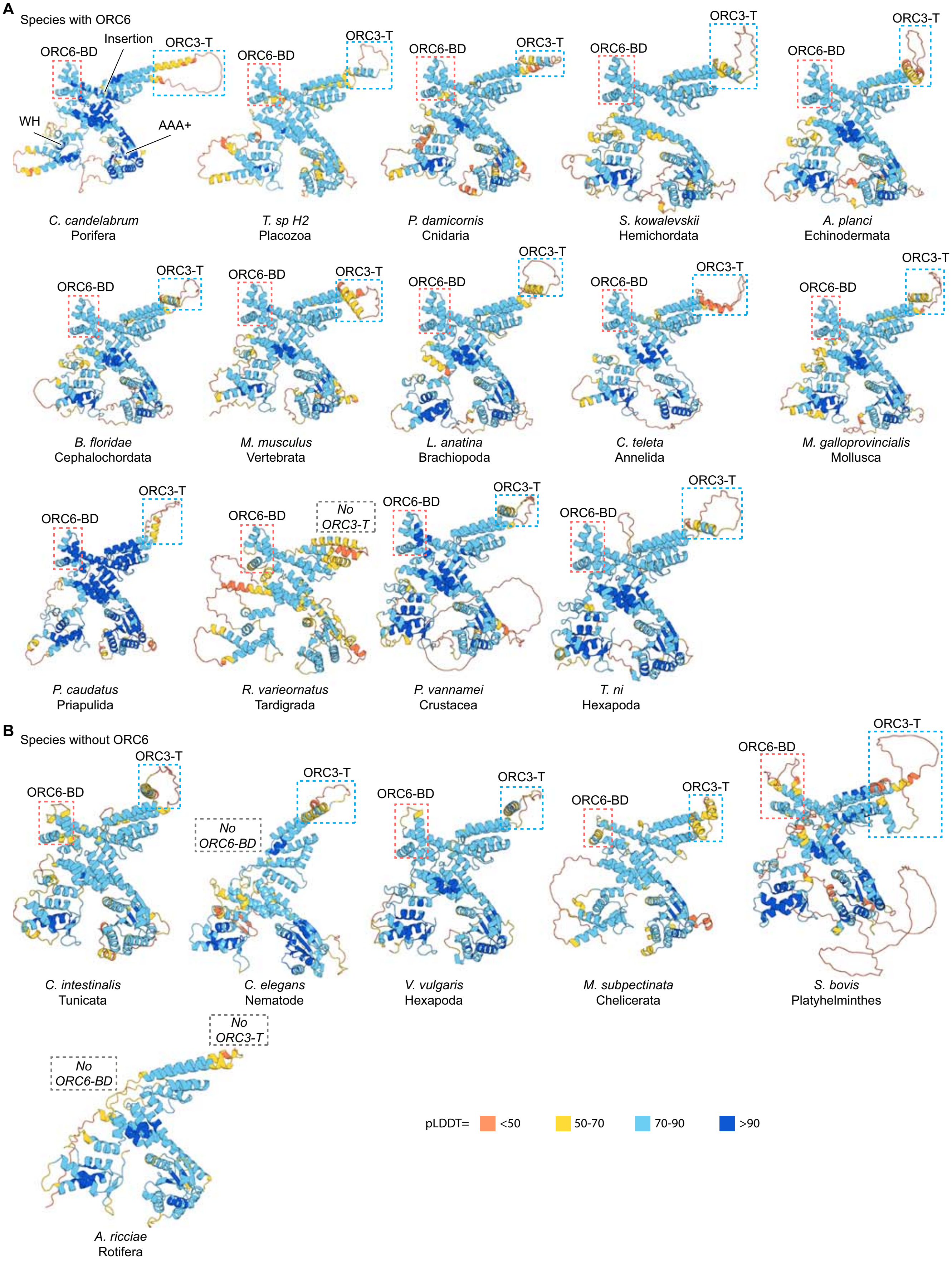
A
Species with ORC6
ORC6-BD
Insertion
ORC3-T
WH
AAA+
C. candelabrum
Porifera
T. sp H2
Placozoa
P. damicornis
Cnidaria
S. kowalevskii
Hemichordata
A. planci
Echinodermata
B. floridae
Cephalochordata
M. musculus
Vertebrata
L. anatina
Brachiopoda
C. teleta
Annelida
M. galloprovincialis
Mollusca
P. caudatus
Priapulida
No ORC3-T
R. varieornatus
Tardigrada
P. vannamei
Crustacea
T. ni
Hexapoda
B
Species without ORC6
C. intestinalis
Tunicata
No ORC6-BD
C. elegans
Nematode
V. vulgaris
Hexapoda
M. subpectinata
Chelicerata
S. bovis
Platyhelminthes
No ORC3-T
No ORC6-BD
A. ricciae
Rotifera
pLDDT=
<50
50-70
70-90
>90

Figure EV2. Example AlphaFold2 predictions of ORC3 orthologs.

(**A**) ORC3 predictions for species with ORC6 and (**B**) without ORC6 orthologs for each phylum/subphylum in our dataset. Models are colored according to pLDDT scores. The ORC6-BD and ORC3 tether regions, if present, are labeled.

 © The Author(s)

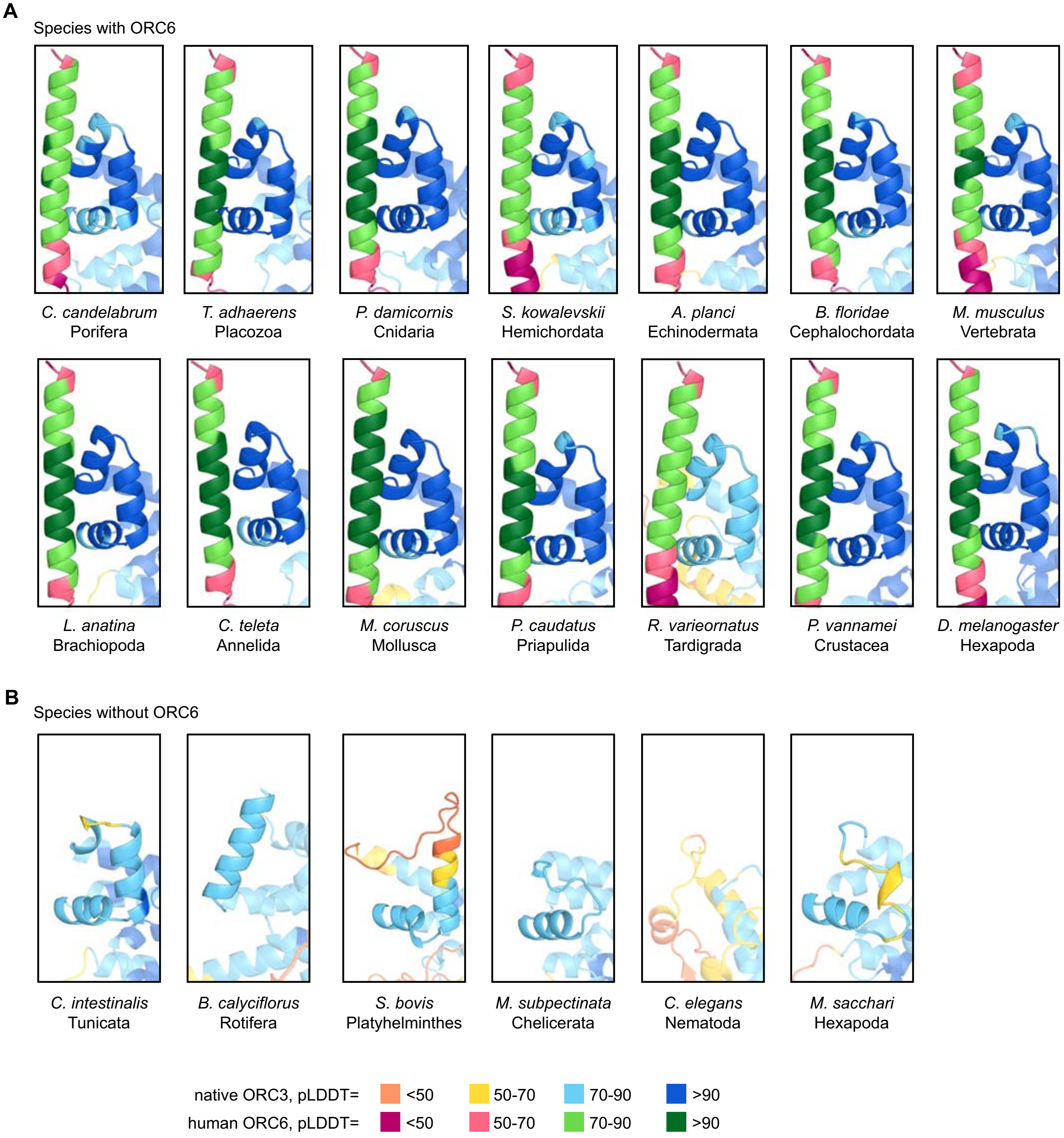

Figure EV3. Example AlphaFold2 Multimer-predicted interfaces between human ORC6 and ORC3 from various metazoan species.

Example AlphaFold2 Multimer predictions between human ORC6 and ORC3 from species (**A**) with ORC6 and (**B**) without ORC6 orthologs for each phylum/subphylum in our dataset. Although all predictions were done with human ORC6 as input sequence, no interactions with ORC3 are predicted in species that have lost ORC6. Views are zoomed on the canonical ORC3-ORC6 interaction interface. Models are colored according to pLDDT scores.

© The Author(s)

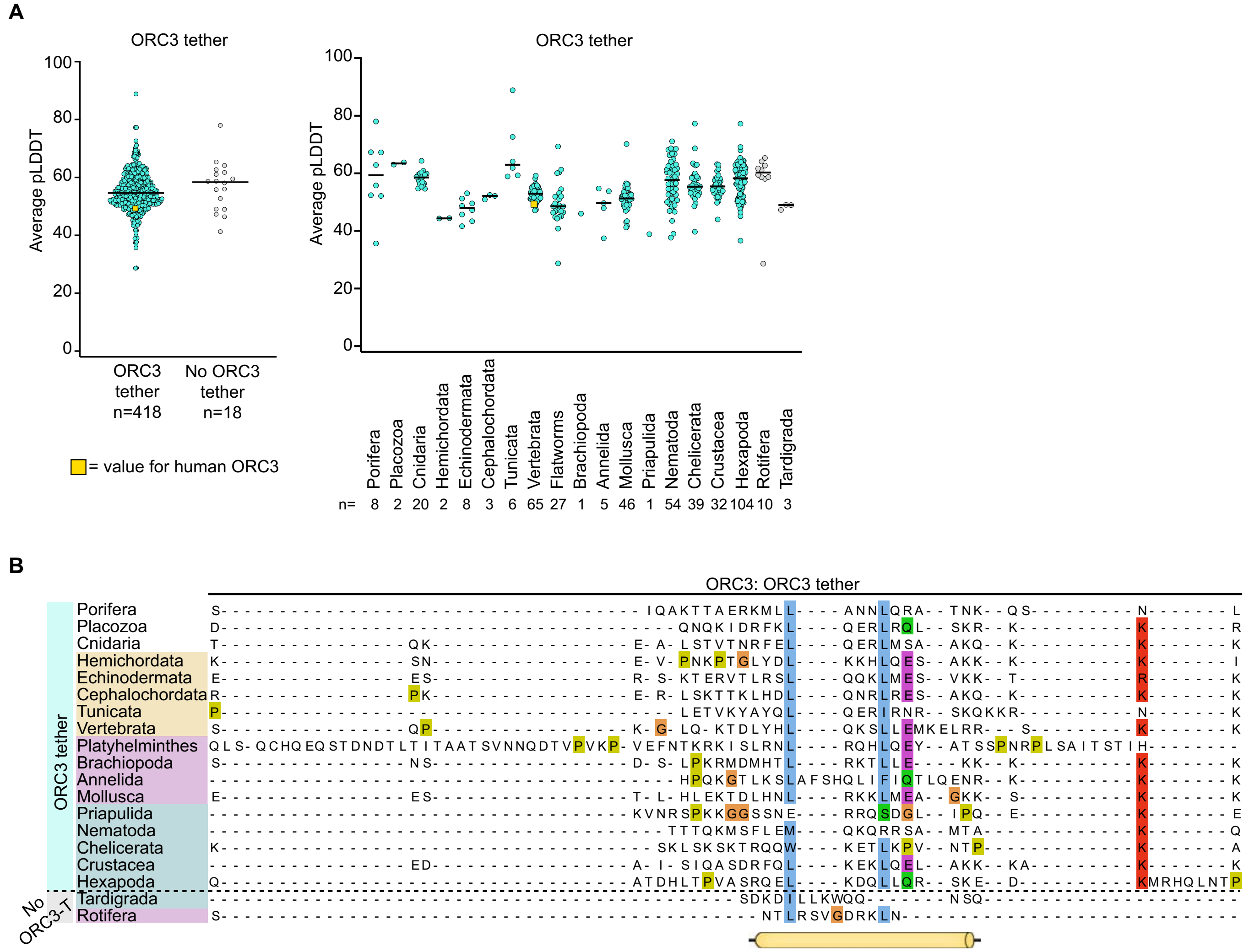

Figure EV4. The ORC3 tether displays moderate structural and sequence conservation.

(**A**) Average pLDDT scores for the ORC3 tether region in AlphaFold2 monomer predictions of ORC3 orthologs across Metazoa. Scores for ORC3 orthologs with and without the ORC3 tether are plotted on the left, and those for individual phyla/subphyla are plotted on the right. Solid black lines represent medians. (**B**) Multiple protein sequence alignment of ORC3 showing the ORC3 tether region. The cylinder below the alignment denotes a predicted α-helix in the tether.

© The Author(s)

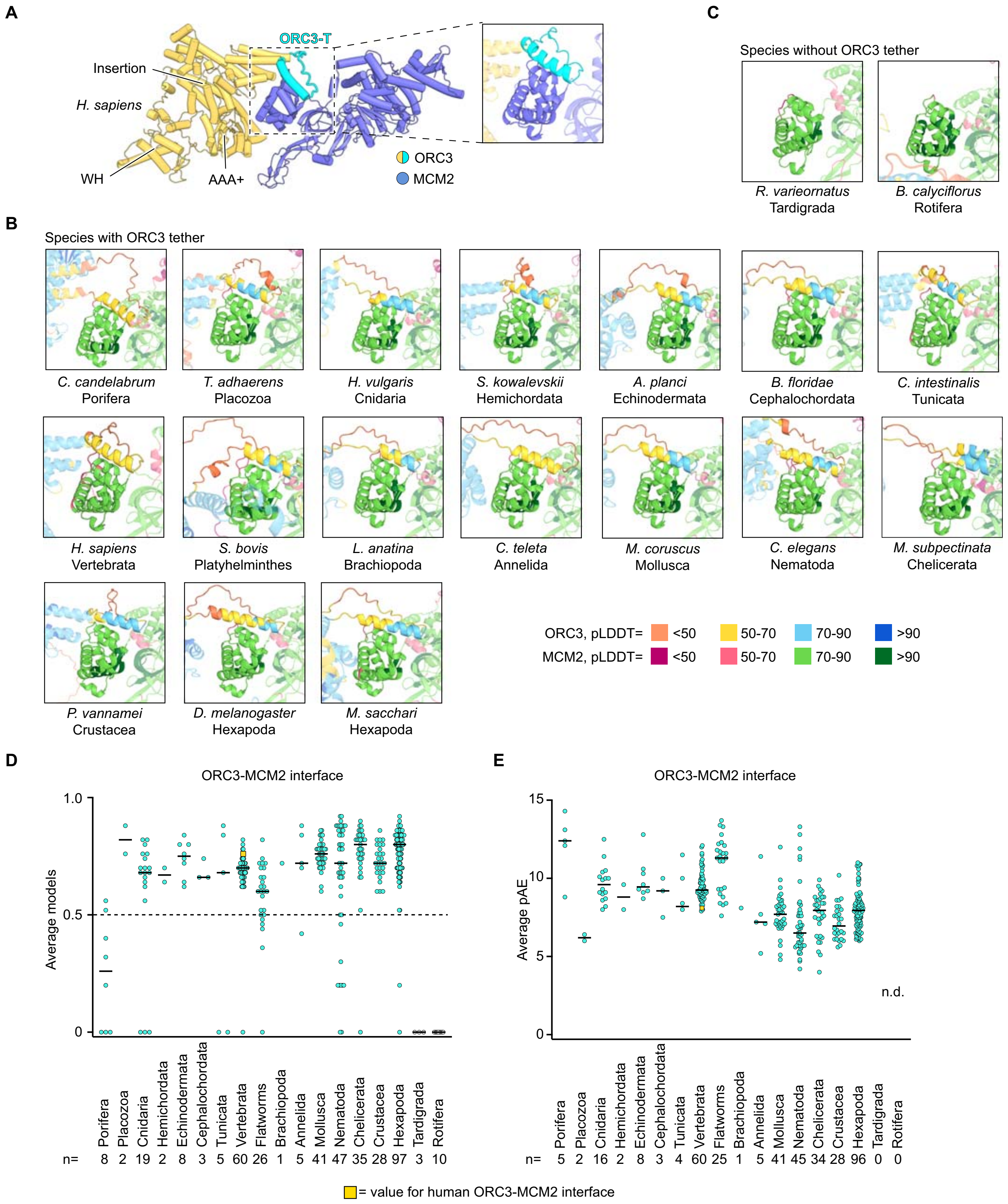
A
ORC3-T
Insertion
H. sapiens
WH
AAA+
ORC3
MCM2
C
Species without ORC3 tether
R. varieornatus
Tardigrada
B. calyciflorus
Rotifera
B
Species with ORC3 tether
C. candelabrum
Porifera
T. adhaerens
Placozoa
H. vulgaris
Cnidaria
S. kowalevskii
Hemichordata
A. planci
Echinodermata
B. floridae
Cephalochordata
C. intestinalis
Tunicata
H. sapiens
Vertebrata
S. bovis
Platyhelminthes
L. anatina
Brachiopoda
C. teleta
Annelida
M. coruscus
Mollusca
C. elegans
Nematoda
M. subpectinata
Chelicerata
P. vannamei
Crustacea
D. melanogaster
Hexapoda
M. sacchari
Hexapoda
ORC3, pLDDT= <50 50-70 70-90 >90
MCM2, pLDDT= <50 50-70 70-90 >90
D
ORC3-MCM2 interface
Average models
1.0
0.5
0
Porifera Placozoa Cnidaria Hemichordata Echinodermata Cephalochordata Tunicata Vertebrata Flatworms Brachiopoda Annelida Mollusca Nematoda Chelicerata Crustacea Hexapoda Tardigrada Rotifera
n= 8 2 19 2 8 3 5 60 26 1 5 41 47 35 28 97 3 10
E
ORC3-MCM2 interface
Average pAE
15
10
5
0
n.d.
Porifera Placozoa Cnidaria Hemichordata Echinodermata Cephalochordata Tunicata Vertebrata Flatworms Brachiopoda Annelida Mollusca Nematoda Chelicerata Crustacea Hexapoda Tardigrada Rotifera
n= 5 2 16 2 8 3 4 60 25 1 5 41 45 34 28 96 0 0
= value for human ORC3-MCM2 interface

Figure EV5. Example AlphaFold2 multimer-predicted interfaces between metazoan ORC3 and MCM2 and associated confidence statistics.

(**A**) AlphaFold2 multimer-predicted interactions between human ORC3 and MCM2. Zoomed view shows interaction between the MCM2 N-terminal domain and the ORC3 tether. (**B**, **C**) Example AlphaFold2 multimer-predicted ORC3-MCM2 interfaces for species with an ORC3 tether (in **B**) and without an ORC3 tether (in **C**). Models are colored according to pLDDT scores. In (**C**), no interaction between ORC3 and MCM2 is predicted by AlphaFold despite providing both sequences in the input. (**D**) *Average models* scores for AlphaFold2 multimer ORC3-MCM2 predictions by taxonomic group. Dotted black line at *average models* = 0.5 marks the confidence cut-off. (**E**) Average interface pAE scores of ORC3-MCM2 interactions by taxonomic group. If no interface was formed, pAE could not be calculated and no data is shown for these datapoints. Solid black lines in (**D**, **E**) are medians. Values of 0 in (**D**) indicate that no canonical ORC3-MCM2 interface was predicted.

© The Author(s)
